# Protein kinase C inhibitor chelerythrine selectively inhibits proliferation of triple-negative breast cancer cells

**DOI:** 10.1038/s41598-017-02222-0

**Published:** 2017-05-17

**Authors:** Wanjun Lin, Jiajun Huang, Zhongwen Yuan, Senling Feng, Ying Xie, Wenzhe Ma

**Affiliations:** State Key Laboratory of Quality Research in Chinese Medicine, Macau University of Science and Technology, Macau, China

## Abstract

Triple-negative breast cancer (TNBC) is a subtype of breast cancer lacking targeted therapy currently. Recent studies imply that protein kinase C may play important roles in TNBC development and could be a specific target. In this study, we evaluated the anti-proliferative activity of PKC inhibitor chelerythrine on a panel of breast cancer cell lines. Chelerythrine selectively inhibited the growth of TNBC cell lines compared to non-TNBC cell lines as demonstrated by *in vitro* cell proliferation assay and colony formation assay, as well as evidenced by *in vivo* xenograft assay. The selective anti-proliferative effect of chelerythrine was associated with induction of apoptosis in TNBC cell lines. We further demonstrated that PKN2, one of the PKC subtypes, was highly expressed in TNBC cell lines, and knocking down PKN2 in TNBC cells inhibited colony formation and xenograft growth. This indicates that PKN2 is required for the survival of TNBC cells, and could be the target mediates the selective activity of chelerythrine. Finally, combination of chelerythrine and chemotherapy reagent taxol showed synergistic/additive effect on TNBC cell lines. Our results suggest chelerythrine or other PKC inhibitors may be promising regimens for TNBC tumors.

## Introduction

Breast cancer is the most common cancer in women worldwide, with an estimated 1.67 million new cases diagnosed and more than half million deaths in 2012^1^. Clinically, based on the expression levels of estrogen receptor (ER), progesterone receptor (PR), and human epidermal growth factor receptor 2 (HER2), breast cancer is classified into subgroups of hormone receptor-positive, HER2-positive, and triple-negative breast cancer^2^. Triple-negative breast cancer (TNBC), characterized by absence of ER/PR and lack of overexpression of HER2, represents approximately 10–15% of all breast cancers^3^.

As TNBC does not respond to either hormonal therapy or anti-HER2 agents, stand chemotherapy is currently the mainstay of systemic medical treatment with this subtype of breast cancer^4^. TNBC initially responds to conventional chemotherapy, however patients frequently show repaid disease relapses^5^ and currently there is no effective treatment after a relapse^6^. In addition, TNBC is more aggressive than other types of breast cancer, which is likely to metastasize to the lungs and brain^7, 8^. So patients with TNBC usually have a poor prognosis and a shorter overall survival compared to other subtypes of breast cancer. New therapies targeting poly (ADP-ribose) polymerase (PARP), epidermal growth factor receptor (EGFR), angiogenesis, mammalian target of rapamycin (mTOR), proteasome, cyclin-dependent kinase (CDK), histone deacetylase (HDAC), Src kinase, Wnt signaling, and phosphatidylinositide 3-kinases (PI3K) are being actively investigated in clinical trials in patients with TNBC^9–11^. But many of these efforts are not reaching the hoped results, and to date, not a single targeted therapy has been approved for the treatment of TNBC. Therefore, new targeted therapies are in urgent needed for patients with TNBC.

One potential target of TNBC is protein kinase C (PKC), which is a serine/threonine protein kinase family of enzymes and plays critical roles in several disease processes including cancer, diabetes, autoimmune diseases, heart failure, Parkinson’s disease, Alzheimer’s disease, and many other important human diseases^12^. An inverse relationship between ER and PKC activity and abundance in human breast cell lines and tumors has been firmly established^13–15^. PKC is also elevated in malignant versus normal breast tissue^16, 17^, and overexpression of PRKCA (PKC*α*) is associated with antiestrogen resistance^18, 19^ and tumor aggressiveness^20^. PRKCA is shown to be a central signaling node and therapeutic target for breast cancer stem cells^21^, which share similar profile of cell surface markers with TNBC^4^. Furthermore, PRKCA is directly associated with TNBC both in cell lines and in patient tumors^22, 23^.

All above findings imply that PKC plays important roles in TNBC and could be a potential specific therapeutic target for TNBC. However, no studies of the anti-cancer effect of PKC inhibitors specifically targeting TNBC have been reported. One of the most specific PKC inhibitors developed is chelerythrine^24^, a natural benzophenanthridine alkaloid isolated from *Chelidonium majus* and possesses diverse pharmacological activities including potent anti-cancer and cytotoxic activities^25, 26^. Here, we report the selective anti-proliferative activity of chelerythrine against TNBC cell lines. Our data suggest that chelerythine or other PKC inhibitors may be promising regimens for TNBC tumors.

## Materials and Methods

### Reagents and antibodies

Chelerythrine and taxol were purchased from Melonepharma (Dalian, China). Trichloroacetic acid (TCA), propidium iodide (PI), Hoechst 33258, DNase-free RNase A, triton X-100, crystal violet and sulforhodamine B (SRB) were obtained from Sigma Aldrich. Antibody sources were as follows: cleaved nuclear poly (ADP-ribose) polymerase (cPARP, Cell Signaling); PRKCA (BD Biosciences); PKN2 (Abcam); β-actin (Sigma Aldrich); horseradish peroxidase-conjugated secondary antibodies (Jackson Laboratory).

### Cell culture

Breast cancer cell lines MDA-MB-231, BT-549, HCC1937, MDA-MB-468, MCF7, ZR-75-1, SK-BR-3 and MDA-MB-453 (Cell Bank of the Chinese Academy of Sciences, Shanghai, China) were cultured in 1640 medium (Gibco) supplemented with 10% FBS (Gibco) and 1% Pen Strep Glutamine (100X, 10,000 Units/ml penicillin, 10,000 mg/ml streptomycin and 29.2 mg/ml L-Glutamine) (Gibco).

### *In vitro* cell proliferation assay (SRB assay)

The anti-proliferative effects of tested chemicals on breast cancer cell lines were assessed by sulforhodamine B (SRB) colorimetric assay as previously described^27^. Briefly, cells were seeded in 96-well plates in a volume of 100 μl/well at densities of 5,000~40,000 cells per well. After overnight incubation at 37 °C in a humidified incubator with 5% CO_2_, 100 μl medium containing chemicals (2 X indicated concentrations) were added. After treatment for 72 hours, attached cells were fixed with 50 μl cold 50% (w/v) trichloroacetic acid (TCA) for 1 hour at 4 °C and then stained with 100 μl 0.4% (w/v) SRB. The absorbency at 515 nm was measured using SpectraMax 190 microplate reader (Molecular Devices) after solubilizing the protein-bound dye with 200 μl 10 mM Tris base solution (pH 10.5). The IC50 value was defined as the concentration required for a 50% reduction in cell growth.

### Colony formation assay

Cells were either treated with 5 μM chelerythrine for various periods of time or selected with puromycin for 3 days after lentivirus transduction. Cells were then washed with PBS, plated in drug-free medium in 6-well plates at densities of 1,000 cells/well and incubated for 7–10 days in the absence of drug. Colonies were stained with 0.2% (w/v) crystal violet in buffered formalin for 10 minutes. The number of colonies was counted.

### Flow-cytometric analysis of cell cycle

Flow cytometric analyses were performed to define the cell cycle distribution after treatment with 5 μM chelerythrine. Cells were harvested, washed twice with PBS, resuspended in 0.5 ml PBS (1,000,000~2,000,000 cells/ml). Then 4 ml 70% ethanol was added and kept at −20 °C for 2 hours to fix the cells. Cells were stained for total DNA content with a solution containing 20 μg/ml propidium iodide, 200 μg/ml DNase-free RNase A, and 0.1% triton X-100 in PBS for 30 minutes at room temperature. Cell cycle distribution was analyzed by flow cytometry (BD Bioscience). The percentage of the total cell population in the four different phases (Sub-G0/G1, G0/G1, S, G2/M) of cell cycle was determined using FlowJo software.

### Flow-cytometric analysis of apoptosis

Cellular apoptosis was analyzed with BD Annexin V: Fitc Apoptosis Detection Kit I (BD Bioscience) by flow cytometry. Briefly cells were plated in 6-well plates (100,000~400,000 cells/well) and treated with chelerythrine. At the indicated time point, cells were harvested, washed twice with cold PBS, and resuspended in 1X Binding Buffer at a concentration of 1,000,000 cells/ml. 100 μl cells were transferred to 1.5 ml conical tube and 5 μl FITC Annexin V and 5 μl propidium iodide were added. The mixture was gently vortexed and incubated at room temperature for 15 minutes in the dark, followed by adding 400 μl 1x Binding Buffer to each tube. Cells were filtered and analyzed by flow cytometry (BD Bioscience) within 1 hour. Total apoptotic cells (FITC Annexin V positive) were counted.

### Assessment of cell morphological changes

Cells were plated in 12-well plates (80,000–200,000 cells/well) then treated with 5 μM chelerythrine for 24 hours. After incubation, cells were collected, washed with PBS and stained with Hoechst 33258 (11.1 μg/ml) in buffered formalin solution containing 5.6% NP-40. Living and apoptotic cells were visualized through DAPI filter of fluorescence inverted microscope (Leica DM2500 Fluorescence Microscope) at ×400 magnification.

### Lentivirus mediated gene knockdown

The following double strand oligos (only sense strands indicated) were cloned into the pLKO.1 plasmid at *Age*I and *EcoR*I sites and sequence verified before use. shPRKCA: 5′-CCGGCGAGGTGAAGGACCACAAATTCTCGAGAATTTGTGGTCCTTCACCTCGTTTTTTG-3′, shPKN2: 5′-CCGGGTCCACGTCAAAGTATGATATCTCGAGATATCATACTTTGACGTGGACTTTTTG-3′. A MISSION non-target shRNA control vector served as the scrambled control (Sigma-Aldrich, SH002). Lentivirus were produced by cotransfection of 293T cells with above constructs and the MISSION packing mix (Sigma-Aldrich) using FuGENE HD transfection reagent (Promega). Cells were incubated with lentivirus for 24 hours before selection with puromycin (Gibco). Gene knockdown was confirmed by western blotting analysis.

### Retrieval of gene expression data from CCLE database

Cancer Cell Line Encyclopedia (CCLE) data on breast cancer cell lines was used to compare mRNA expression of TNBC and non-TNBC cells^28^. The log_2_ radio of TNBC cell lines (n = 26) versus non-TNBC cell lines (n = 30) was analyzed using GENE-E and Prism software.

### Quantitative real-time PCR

Cellular mRNA was purified by binding to poly(dT) magnetic beads (Dynal) and reverse transcribed using SuperScript III (Invitrogen) as described by the manufacturer. Quantitative real-time PCR was performed in duplicates three times by using SYBR Green (Molecular Probes) on the ViiA™ 7 Real-Time PCR System (Applied Biosystems). Data were expressed as relative mRNA levels normalized to the eukaryotic translation initiation factor (EIF3S5 or TIF) expression level in each sample. The primer sequences can be obtained upon request.

### Western blotting

Protein samples were prepared by adding RIPA buffer with protease inhibitor cocktail (Roche) to cells and diluted in SDS-PAGE protein sample buffer. Samples were heated for 5 minutes at 95 °C before fractionation on SDS-polyacrylamide gels. The proteins were then transferred to Immobilon P (Millipore) and incubated with primary antibodies overnight at 4 °C. The membranes were then washed with TBST and incubated with the appropriate horseradish peroxidase-conjugated secondary antibodies at room temperature. Proteins were visualized with SuperSignal West Dura Extended Duration Substrate or SuperSignal West Pico Chemiluminescent Substrate (Thermo Scientific).

### Xenograft assay

4–6 weeks old female nude mice were injected subcutaneously of 2 × 10^6^ cells resuspended in 100 μl PBS into both hind limbs. Chelerythrine was injected intraperitoneally at a dose of 5 mg/kg at 3–4 days intervals. Tumors were measured by digital caliper and volumes were calculated using the following equation, volume = (width^2^ × length)/2. All procedures were carried out in accordance with guidelines by Division of Animal Control and Inspection of the Department of Food and Animal Inspection and Control of Macau and were approved by the Animal Care and Use Committee (ACUC) of the Macau University of Science and Technology.

### Dual-Drug combination assay

Breast cancer cell lines were plated in 96-well plates (5,000–40,000 cells/well), treated with various concentrations of chelerythrine and taxol, either alone or in combination for 72 hours. Cell number was determined by SRB assay. And the combination index (CI) score was calculated using Compusyn software^29^. The effects of drug combination were determined based on the CI values. CI < 0.9, synergy; 0.9 < CI < 1.1, additive effect; CI > 1.1 antagonism^30^.

### Statistical analysis

Statistical analysis was performed using Microsoft Excel. Data are shown as mean ± SD and the statistical significance was determined by two-tailed Student’s *t* test.

## Results

### Chelerythrine selectively inhibits proliferation of TNBC cell lines *in vitro*

**Figure 1:**
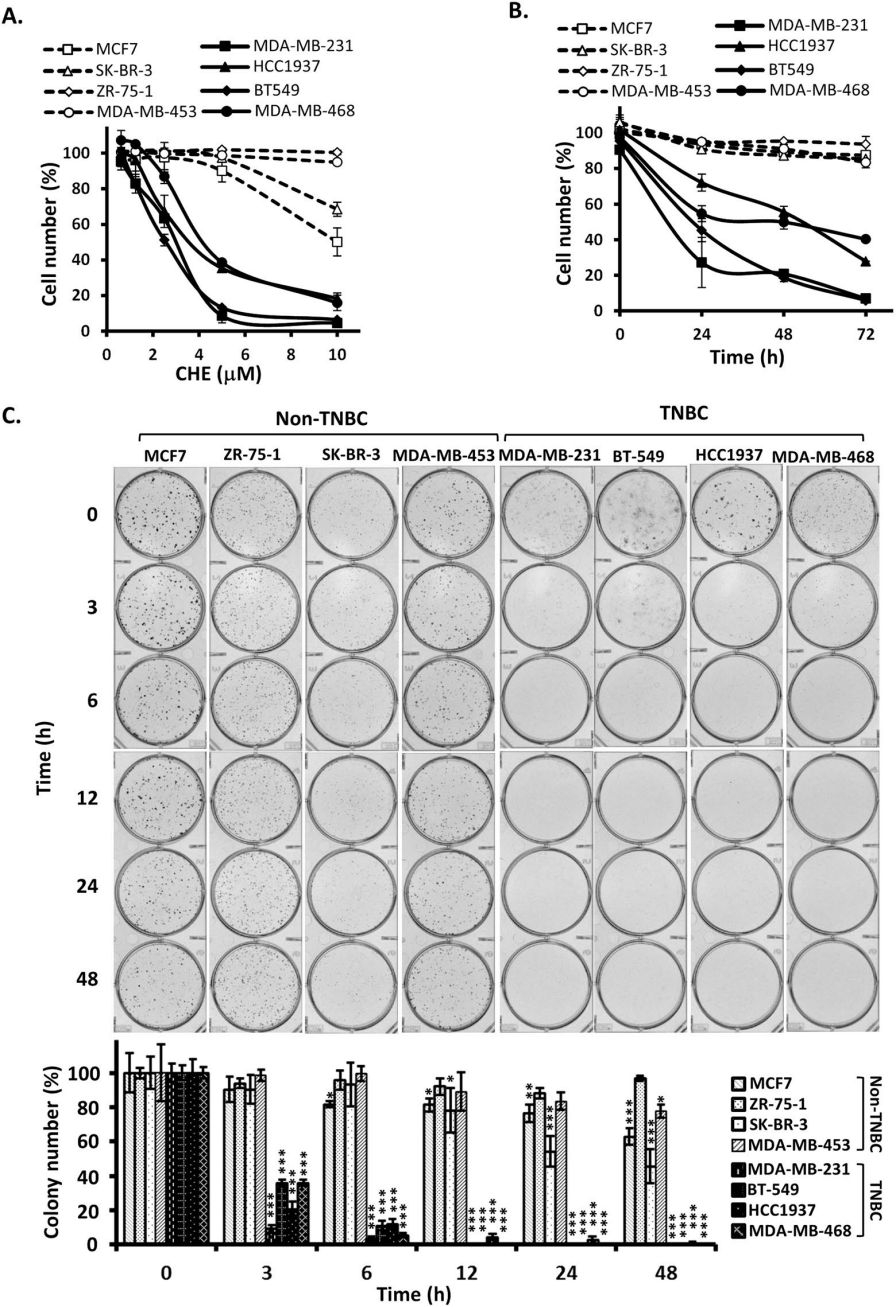
Chelerythrine selectively inhibits proliferation of TNBC cell lines *in vitro*. Four non-triple-negative breast cancer (Non-TNBC) cell lines (MCF7, ZR-75-1, SK-BR-3, MDA-MB-453) and four triple-negative breast cancer (TNBC) cell lines (MDA-MB-231, BT549, HCC1937 and MDA-MB-468) were used to determine the growth inhibition effect of chelerythrine (CHE). (**A**) Dose effect of chelerythrine treatment (72 hours) on the proliferation of non-TNBC cell lines compared with TNBC cell lines. The cell number at each chelerythrine concentration is represented as a percentage of control (no chelerythrine treatment). Average values are from three independent experiments performed in duplicate (n = 3). (**B**) Time course of chelerythrine treatment (5 μM) on the proliferation of non-TNBC cell lines compared with TNBC cell lines. The cell number at each time point is represented as a percentage of control (no chelerythrine treatment). Average values are from three independent experiments performed in duplicate (n = 3). (**C**) Colony formation after treatment with chelerythrine (5 μM) for indicated times. Representative colony formation assay plates are shown, which were quantified by counting colony number (n = 4). Data are shown as mean ± SD. P-values determined by Student’s t-test compared to control. *P < 0.05; **P < 0.01; ***P < 0.001.

To test *in vitro* anti-proliferative activity of chelerythrine on breast cancer cells, four TNBC cell lines (MDA-MB-231, BT-549, HCC1937 and MDA-MB-468) and four non-TNBC cell lines (MCF7, ZR-75-1, SK-BR-3 and MDA-MB-453) were treated with a serials of chelerythrine concentrations ranging from 0.625 μM to 10 μM for 72 hours. As shown in Fig. 1A and Table 1, chelerythrine inhibited the growth of TNBC cell lines dose-dependently, with IC50 values of 2.6 μM to 4.2 μM respectively. On the contrary, the tested non-TNBC cell lines were relative resistant to chelerythrine treatment compared to TNBC cell lines. The IC50 values of all four non-TNBC cell lines were greater than 10 μM, and two of the four cell lines, MDA-MB-453 and ZR-75-1, did not show any inhibitory effect even at the highest concentration tested. When treated with 5 μM of chelerythrine, all four TNBC cell lines demonstrated significant reduction of cell growth in a time dependent manner, while the four non-TNBC cell lines showed much less inhibition of cell growth at each time point (Fig. 1B). So TNBC cells lines are more sensitive to chelerythrine treatment compared to non-TNBC cell lines in a dose- and time-dependent manner. The selective anti-proliferative activity of chelerythrine on TNBC cells were further confirmed by colony formation assay (Fig. 1C). Colony formation ability of TNBC cells were dramatically decreased after treatment with chelerythrine for 3 hours and almost no cells grew into colonies after 6 hours, while non-TNBC cells reserved largely of their colony formation ability even after treatment for 48 hours.

**Table 1:** Mean IC50 values (μM) of chelerythrine on breast cancer cell lines.

Non-TNBC				TNBC			
MCF7	ZR-75-1	SK-BR-3	MDA-MB-453	MDA-MB-231	BT-549	HCC1937	MDA-MB-468
>10	>10	>10	>10	3.0	2.6	3.6	4.2

### Chelerythrine selectively induces apoptosis in TNBC cell lines

Cell cycle arrest is the key cellular event contributing to reduced proliferation, so we first analyzed the effect of chelerythrine on cell cycle progression. Consistent with the unnoticeable cell growth inhibition of 5 μM chelerythrine on non-TNBC cells (Fig. 1B and C), the cell cycle distribution of these non-TNBC cells were basically unchanged with chelerythrine treatment (5 μM) for 24 hours (Supplementary Figure 1A and B). There were also no obvious changes in TNBC cell lines MDA-MB-231 and BT-549, marginal but significant increase of the percentage of cells in G0/G1 phase in HCC1937 cells, and marked increase in G2/M phase in MDA-MB-468 cells (Supplementary Figure 1A and B). So chelerythrine affect cell cycle distribution is cell line dependent, indicating other mechanisms existed for its selectivity on inhibition of TNBC cell growth. It is notable that a substantial proportion of nuclei had a sub-G0/G1 DNA content characteristic of apoptosis upon chelerythrine treatment in all TNBC cell lines, which implies induction of apoptosis could be the cause of selectively anti-proliferative effect of chelerythrine against TNBC cells.

**Figure 2:**
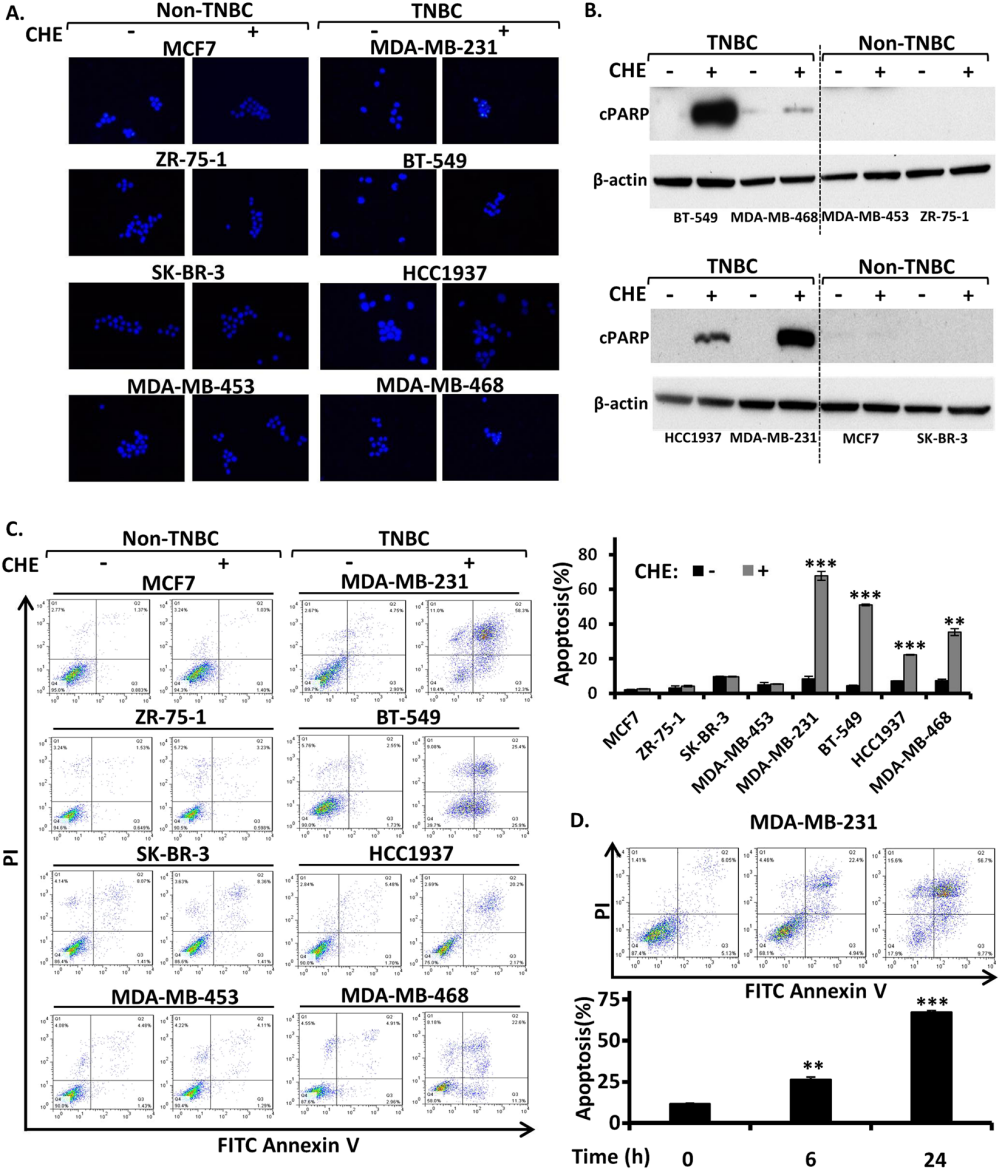
Chelerythrine selectively induces apoptosis in TNBC cell lines. Four non-triple-negative breast cancer (Non-TNBC) cell lines (MCF7, ZR-75-1, SK-BR-3, MDA-MB-453) and four triple-negative breast cancer (TNBC) cell lines (MDA-MB-231, BT549, HCC1937 and MDA-MB-468) were treated with chelerythrine (CHE, 5 μM) for 24 hours. (**A**) Visualization of apoptotic morphological changes by fluorescent microscope with Hoechst 33258 staining. Reprehensive pictures are shown (400x). (**B**) Western blotting analysis of apoptosis marker cleaved nuclear poly (ADP-ribose) polymerase (cPARP). (**C**) Representative contour diagrams of FITC Annexin V/PI flow cytometry analysis of cells. Fractions of apoptotic cells were quantified. Average values are from three independent experiments (n = 3). (**D**) TNBC cell line MDA-MB-231 was treated with chelerythrine (CHE, 5 μM) for 0, 6 and 24 hours. Apoptosis was analyzed by flow cytometry with Annexin V/PI staining. Representative contour diagrams and quantified data are shown. Average values are from three independent experiments (n = 3). Data are shown as mean ± SD. P-values determined by Student’s t-test compared to control. **P < 0.01; ***P < 0.001.

Chelerythrine has been reported to induce apoptosis in lymphoma cells^31^, osteosarcoma cells^32^, squamous cell carcinoma lines^33^ and melanoma cells^34^. So we evaluated whether apoptosis is accounted for the selective anti-proliferative activity of chelerythrine on TNBC cells. As expected, chelerythrine indeed caused chromatin condensation and nuclear fragmentation, the typical morphological characteristics of apoptosis, in TNBC cells but not in non-TNBC cells as visualized by Hoechst staining (Fig. 2A). This was further evidenced by the detection of the cleaved nuclear poly (ADP-ribose) polymerase (cPARP), a marker of apoptosis, in TNBC cell lines compared with non-TNBC cell lines after treatment with chelerythrine by western blotting (Fig. 2B). We then quantified the apoptotic cell fractions by flow cytometry with annexin V and propidium iodide double staining after 24 hours incubation with chelerythrine. The mean percentages of apoptotic cells after chelerythrine treatment of TNBC cell lines MDA-MB-231, BT-549, HCC1937 and MDA-MB-468 were 67.8%, 51.0%, 22.2% and 35.3% respectively, whereas all four non-TNBC cell lines remained viable under the same treatment (Fig. 2C). The induction of apoptosis with chelerythrine treatment in TNBC cells was also time-dependent as exemplified by MDA-MB-231 cells (Fig. 2D). In short, the distinct apoptosis induction activity of chelerythrine on TNBC cells versus non-TNBC cells contributes to its selective anti-proliferative activity on breast cancer cells.

### PKN2, one of PKC isozymes, is highly expressed in TNBC cells

Chelerythrine is known as a highly specific inhibitor of protein kinase C, a family of serine/threonine protein kinase enzymes consisting of at least 12 members that can be categorized into four subgroups specified by their divergent regulatory domains^35^. All the PKC isozymes share a highly conserved kinase/catalytic domain that chelerythrine can interact with and thus inhibits their activities^36^. So we reasoned that one or more PKC isozymes are highly expressed and required for proliferation in TNBC cells compared to non-TNBC cells. By inhibiting the PKC subtype(s), chelerythrine selectively suppresses the growth of TNBC cells.

Among all 12 isotypes of PKC, PRKCA, is the only isoform that has been associated with TNBC both in cell lines and in patient tumors^22, 23^. So we first checked the expression levels of PRKCA in breast cancer cell lines. However, PRKCA was only highly expressed in two of the TNBC cell lines, MDA-MB-231 and BT-549, as manifested by both the mRNA level (Supplementary Figure 2A) and the protein level (Supplementary Figure 2B). Which was consistent with the fact that only in these two cell lines, colony formation was significantly decreased when PRKCA was knocked down by lentivirial-mediated shRNA (Supplementary Figure 2C and D). The data implies that the selective anti-proliferative activity of chelerythrine on TNBC cell lines cannot be fully explained by the expression levels of PRKCA.

**Figure 3:**
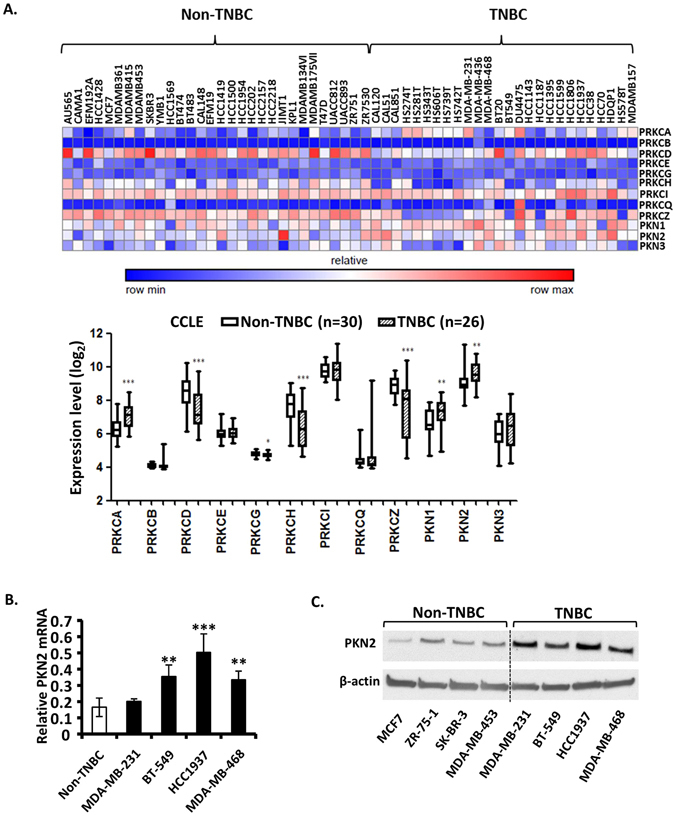
PKN2, one of PKC isozymes, is highly expressed in TNBC cells. (**A**) Relative mRNA expression levels (log_2_) of 12 PKC isozymes in human breast cancer cell lines from Cancer Cell Line Encyclopedia (CCLE) database (n = 56). The heatmap represents color-coded expression levels of differentially expressed PKC isozymes in human breast cancer cell lines. The color scale ranges from saturated blue for the minimum to saturated red for maximum. Average values in the graph are the mean value of all the non-TNBC cell lines (n = 30) and TNBC cell lines (n = 26). (**B**) Quantitative real-time PCR analysis of PKN2 in four non-TNBC cell lines (average value of four cell lines is shown) compared with four TNBC cell lines (individual value of each cell line is shown). Average values are from three independent experiments performed in duplicate (n = 3). (**C**) Western blotting analysis of PKN2. Data are shown as mean ± SD. P-values determined by Student’s t-test compared to non-TNBC cell lines. *P < 0.05; **P < 0.01; ***P < 0.001.

Since chelerythrine is a pan PKC inhibitor, we then proposed that other PKC subtypes may also be essential in TNBC cell proliferation. We retrieved gene expression profile data of 12 PKC isozymes in 56 human breast cancer cell lines, including 26 TNBC cell lines and 30 non-TNBC cell lines, from the Cancer Cell Line Encyclopedia (CCLE) database^28^. Besides PRKCA, we identified two other PKC subtypes, PKN1 and PKN2, which were significant higher in TNBC cell lines than in non-TNBC cell lines (Fig. 3A). PKN1 mRNA was significantly higher in TNBC cell lines, however the protein level was not (data not shown). PKN2 mRNA level was higher in all four TNBC cell lines than non-TNBC cells even though it was not significant in MDA-MB-231cells (Fig. 3B). It was confirmed by western blotting analysis of PKN2 proteins in breast cancer cell lines tested in this study (Fig. 3C). It is notable that PKN2 is one of the highest expressed PKC isoforms in breast cancer cell lines (Fig. 3A), implicating the importance in tumorigenesis. So we focused on PKN2 as the main target of chelerythrine in this study.

### PKN2 expression is essential for TNBC cell growth

**Figure 4:**
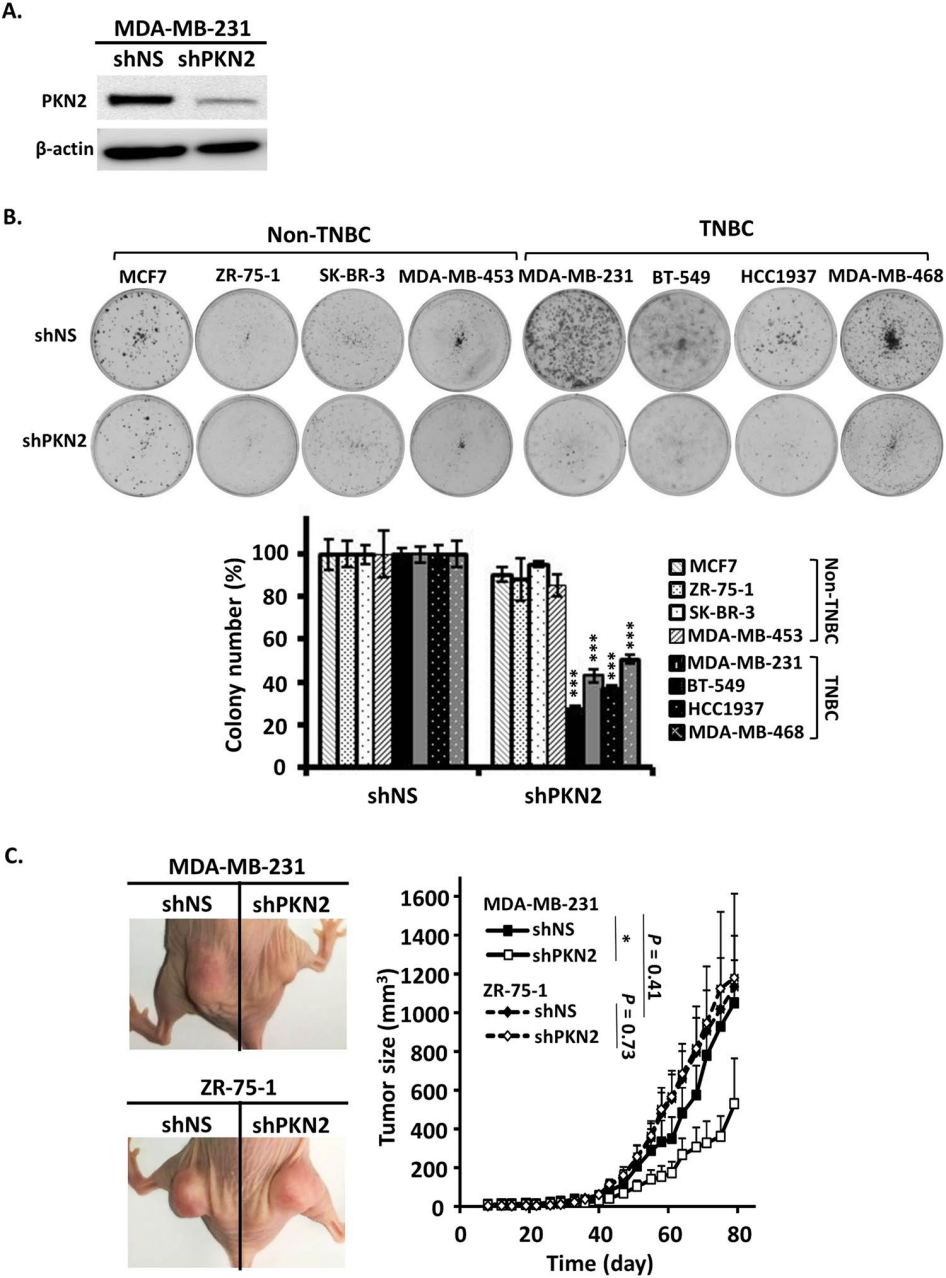
PKN2 expression is essential for TNBC cell growth. Breast cancer cell lines were transduced with either nonspecific shRNA (shNS) or PKN2-specific shRNA (shPKN2) lentivirus and selected with puromycin for 3 days. (**A**) Western blotting analysis of PKN2 after lentiviral knockdown as exemplified in MDA-MB-132 cells. (**B**) Effect of PKN2 knockdown on colony formation of non-TNBC and TNBC cell lines. Representative colony formation assay plates are shown, which were quantified by counting colony number (n = 4). (**C**) Effect of PKN2 knockdown on xenograt formation of TNBC MD-MB-231 cells and non-TNBC ZR-75-1 cells. Left panel, xenograft pictures of one representative mouse for each cell line. Right panel, tumor growth curve over time (n = 9). P values were calculated on day 79 after cell injection. Data are shown as mean ± SD. P-values determined by Student’s t-test. *P < 0.05; ***P < 0.001.

To test whether PKN2 was required to support the growth of TNBC cells, we stably introduced shRNA targeted against PKN2 (Fig. 4A) and examined colony formation of TNBC cell lines and non-TNBC cell lines. As expected, knocking down PKN2 in TNBC cell lines significantly decreased colony formation (Fig. 4B). In contrast, there were no obvious changes in colony formation in all four non-TNBC cell lines (Fig. 4B).

We next sought to test whether increased PKN2 expression in TNBC cell lines is also necessary for cell growth by xenograft assay. TNBC cell line MDA-MB-231 and non-TNBC cell line ZR-75-1 were first transduced with either nonspecific shRNA or PKN2-specific lentivirus and then were injected into contra-lateral hind limbs of athymic nude mice with equal number of cells. PKN2 deficiency did not change xenograft formation of non-TNBC cell line ZR-75-1 but significantly decreased the growth of TNBC cell line MDA-MB-231 (Fig. 4C).

Both the *in vitro* colony formation assay and the *in vivo* xenograft assay after PKN2 knockdown underscored its important roles in supporting TNBC cell growth. So inactivation of PKN2 by chelerythrine selectively inhibits TNBC cell growth.

### Chelerythrine inhibits xenograft formation of TNBC cells and enhances chemotherapy activity of taxol

**Figure 5:**
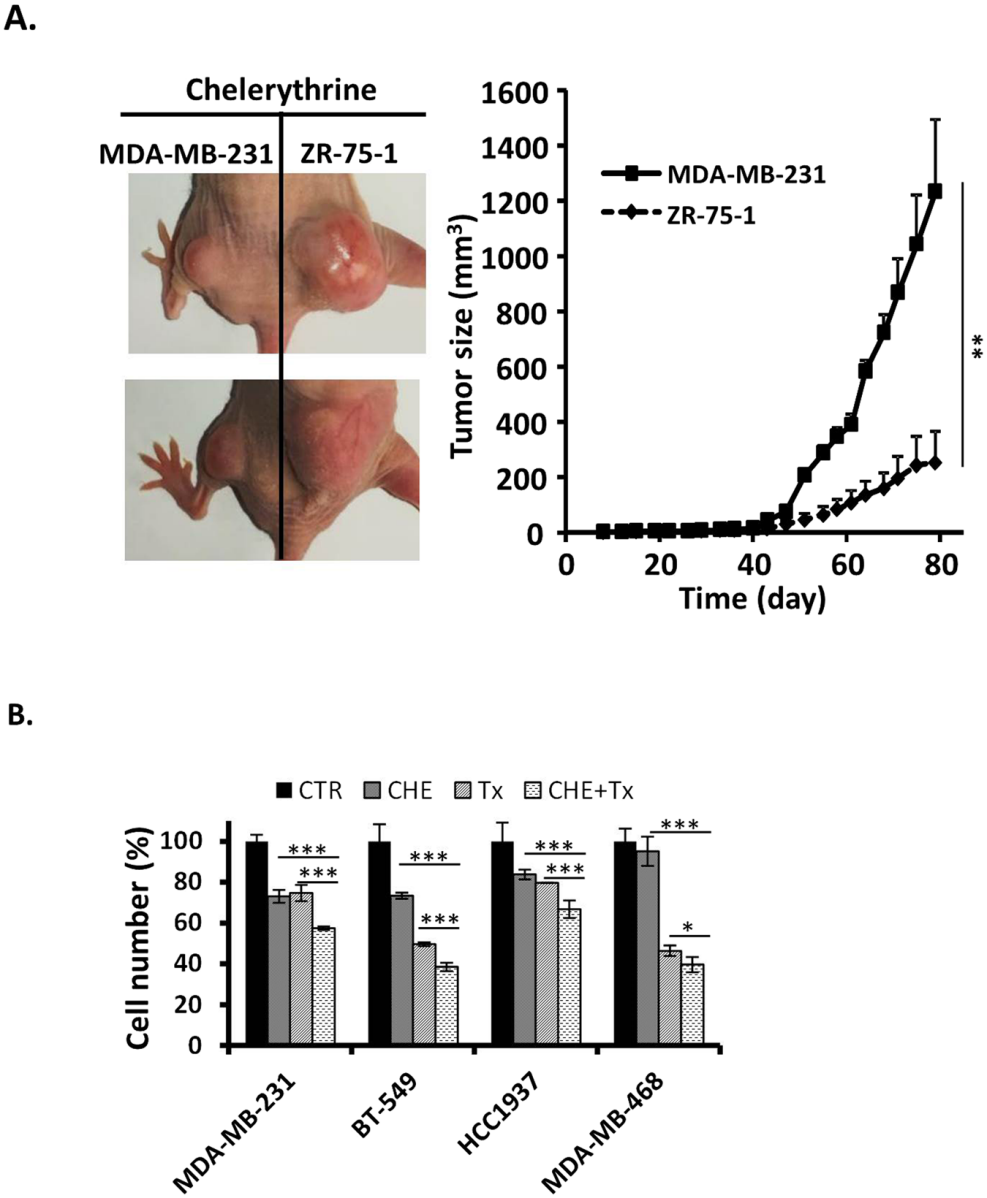
Chelerythrine inhibits xenograft formation of TNBC cells and enhances chemotherapy activity of taxol. (**A**) TNBC cell MDA-MB-231 and non-TNBC cell ZR-75-1 were injected subcutaneously into each hind limbs of nude mice and chelerythrine was administrated intraperitoneally at a dose of 5 mg/kg at 3–4 days intervals. Left panel, xenograft pictures of two representative mice. Right panel, tumor growth curve over time (n = 9). P values were calculated on day 79 after cell injection. (**B**) TNBC cell lines (MDA-MB-231, BT-549, HCC1937 and MDA-MB-468) were treated with either chelerythrine (CHE, 2 μM) or taxol (Tx, 10 nM) only, or a combination of dual drugs (CHE, 2 μM + Tx, 10 nM). The cell number is represented as a percentage of control (CTR, no drug treatment). Average values are from three independent experiments performed in duplicate (n = 3). Data are shown as mean ± SD. P-values determined by Student’s t-test. *P < 0.05; **P < 0.01; ***P < 0.001.

Chelerythrine is able to selectively inhibit *in vitro* TNBC cell growth as shown above, highlighting the possible application of the reagent for TNBC tumors as a novel targeted therapy. We next tested its *in vivo* activity in xenograft tumor study. The xenograft tumor growth rates were similar between TNBC cell line MDA-MB-231 and non-TNBC cell line ZR-75-1 (Fig. 4C). When treated with chelerythrine, the growths of MDA-MB-231 tumors were significantly suppressed compared to ZR-75-1 tumors (Fig. 5A).

Currently there is no targeted therapy available for TNBC tumors and chemotherapy is still the standard regime for TNBC patients. We, therefore, examined the effect of chelerythrine in combination with chemotherapy agent taxol on the proliferation of TNBC cells. Treatment with dual drug combination significantly decreased cell proliferation than either drug given individually in all four TNBC cell lines (Fig. 5B). Furthermore, dual treatment with both chelerythrine and taxol had either additive effect or synergistic effect as manifested by combination index (CI) values at ED50 ranging from 0.75190 to 1.13763 (Table 2). Our data suggest that chelerythrine enhances the anti-proliferative effect of taxol on TNBC cells, and combination of PKC inhibitors with chemotherapy agents may be a promising regime for TNBC treatment.

**Table 2:** Combination index (CI) values at ED50 for the combination of chelerythrine and taxol.

MDA-MB-231	BT-549	HCC1937	MDA-MB-468
1.00098	1.13765	0.75190	1.08958

## Discussion

In summary, we have shown that chelerythrine selectively inhibits proliferation of TNBC cell lines both time- and dose-dependently, which was consistent with dramatic decrease of colony formation in TNBC cell lines treated with chelerythrine. We further demonstrated that the selective anti-proliferative activity of chelerythrine on TNBC cells is resulted from substantial induction of apoptosis. Finally, chelerythrine significantly inhibited *in vivo* xenograft formation and increased the cytotoxic effect of chemotherapy agent taxol against TNBC cell lines. Our data suggest chelerythrine might be a promising regimen selectively targeting TNBC tumors which warranted further study.

We found PKN2 is the only PKC isoform highly expressed in all TNBC cell lines compared to non-TNBC cell lines tested in this study (Fig. 3B and C), and suppression of PKN2 significantly decreased colony formation (Fig. 4B) and xenograft growth (Fig. 4C) in TNBC cells, indicating PKN2 is the target of chelerythrine responsive for its selective activity on TNBC cells. PKN2 plays important roles in cellular processes, including cell cycle progression^37^, actin cytoskeleton assembly^38^, cell adhesion^39^, tumor cell migration and invasion^40^ and apoptosis^41, 42^. Our findings indicate that PKN2 is required for TNBC cells growth, which could be a potential diagnostic biomarker or a therapeutic target for TNBC. However, the hypothesis was only tested in limited cell lines in this study. It is needed to be further verified in more breast cancer cell lines and clinical specimens.

We also showed that PRKCA, the alpha isotype of PKC, was overexpressed in two TNBC cell lines tested in this study (Supplementary Figure 2A and B), and knocking down PRKCA suppressed colony formation in the two cell lines (Supplementary Figure 2D). Our data indicates that PRKCA is required for the growth in these two TNBC cell lines, which is consistent with the findings that PRKCA is associated with TNBC both in cell lines and in patient tumors^22, 23^. So even though we identified PKN2 as the possible target of cherethrine responsive to the selective activity against TNBC cells, we still cannot rule out other PKC isozymes, like PRKCA, also play roles in the phenomenon observed in this study. Further studies are needed to clarify the functions of each individual PKC isoform in TNBC tumor development. Combined inhibition of PKC isoforms will be a more precise approach to TNBC tumors based on expression and function of certain PKC isozymes. In fact, there is still a long way to reach the goal, as rare PKC isozyme-specific inhibitors are available^43, 44^. However, simultaneously inhibiting all isozymes by pan PKC inhibitors, like chelerythrine, is still worth to try on TNBC tumors. As we showed greater activities of chelerhthrine on cell growth (Fig. 1) and induction of apoptosis (Fig. 2) in TNBC cell lines overexpressing both PKN2 and PRKCA (MDA-MB-231 and BT-549) in general.

We have also shown that chelerythrine selectively induces apoptosis in TNBC cell lines tested in this study. PKN2 is rapidly and specifically cleaved by caspase-3 within its regulatory domain, during the induction of apoptosis^41^. And the cleaved C-terminal peptide of PKN2 binds to Akt and down regulates its kinase activity, resulting in the amplification of pro-apoptotic signalling in the cell^42^. PRKCA exhibits anti-apoptotic function by suppressing p53 dependent activation of IGFBP3^45^ or by phosphorylation of Bcl2^46^. It will be interesting to test whether chelerythrine induce apoptosis through regulation of these pathways. On the other hand, chelerythrine has been reported inducing apoptosis by inhibiting anti-apoptotic protein Bcl-2^47^, by dephosphorylation of ERK and Akt^48^, by activation of p53^49^, by inhibiting the expression of cytoprotective proteins HSF1 and hsp70^31^, by inducing reactive oxygen species (ROS)^50, 51^, and so on. It will also be interesting to test whether inhibition of PKC, especially PKN2 and PRKCA isoforms, either individually or simultaneously, leading to apoptosis through these pathways.

Most of breast cancer survivors died from noncancer causes, especially for those 50 years and older. And among these causes, cardiovascular disease (CVD) represents the greatest single factor, accounting for approximately 35% of noncancer mortality^52^. CVD caused death is associated with breast cancer treatments, like radiotherapy^53^, HER2 targeted therapy^54^, and chemotherapy^55^. Compared to non-TNBC patients, TNBC patients have a higher prevalence of metabolic syndrome and obesity^56–59^, both are well known as CVD risk factors. While PKC activation is well documented in CVD developmet^60, 61^, and chelerythrine has been reported to improve diabetes induced endothelial dysfunction in rats^62^. All these evidences may add to the value of chelerythrine for TNBC treatment. Which could benefit the patients not only by inhibiting tumor growth, but also by improving CVD complications.

## Electronic supplementary material

Supplementary Information
