# Supplementary material for: Protein kinase C inhibitor chelerythrine selectively inhibits proliferation of triple-negative breast cancer cells
Source: Sci Rep. 2017 May 17;7:2022. doi: 10.1038/s41598-017-02222-0 (PMC5435721; doi:10.1038/s41598-017-02222-0)

SUPPLEMENTAL INFORMATION

Protein kinase C inhibitor chelerythrine selectively inhibits proliferation of triple-negative breast cancer cells

Wanjun Lin*, Jiajun Huang*, Zhongwen Yuan, Senling Feng, Ying Xie and Wenzhe Ma

State Key Laboratory of Quality Research in Chinese Medicine, Macau University of Science and Technology, Macau, China

*These authors contributed equally to this work

Corresponding author:
Wenzhe Ma
State Key Laboratory of Quality Research in Chinese Medicine, MUST
Building H, Rm. 718e
Avenida Wai Long, Taipa, Macau, China
Email: wzma@must.edu.mo
Tel: +853-88972462
Fax: +853-28825886

Supplementary Figure Legends

Supplementary Figure 1. Chelerythrine differentially induces cell cycle arrest in breast cancer cell lines. Four non-triple-negative breast cancer (Non-TNBC) cell lines (MCF7, ZR-75-1, SK-BR-3, MDA-MB-453) and four triple-negative breast cancer (TNBC) cell lines (MDA-MB-231, BT549, HCC1937 and MDA-MB-468) were treated with chelerythrine (CHE, 5 μM) for 24 hours. **(A).** Representative cell cycle distributions analyzed by flow cytometry. **(B)**. Percentages of the total cell population in the four different phases of cell cycle (Sub-G0/G1, G0/G1, S, and G2/M) were determined using FlowJo software. Average values are from three independent experiments (n = 3). Data are shown as mean $\pm$ SD. P-values determined by Student's t-test. **, P<0.01; ***, P<0.001.

Supplementary Figure 2. PRKCA is not overexpressed and required for colony formation in all TNBC cell lines. (A). Quantitative real-time PCR analysis of PRKCA in four non-TNBC cell lines (average value of four cell lines is shown) compared with in four TNBC cell lines (individual value of each cell line is shown). Average values are from three independent experiments performed in duplicate (n = 3). **(B).** Western blotting analysis of PRKCA protein. **(C)**. Western blotting analysis of PRKCA after lentiviral knockdown as exemplified in MDA-MB-231 cells. (D) Effect of PRKCA knockdown on colony formation of non-TNBC and TNBC cell lines. Representative colony formation assay plates are shown, which were quantified by counting colony number (n = 4). P-values determined by Student's t-test. ***, P<0.001.

Supplementary Figure 1.

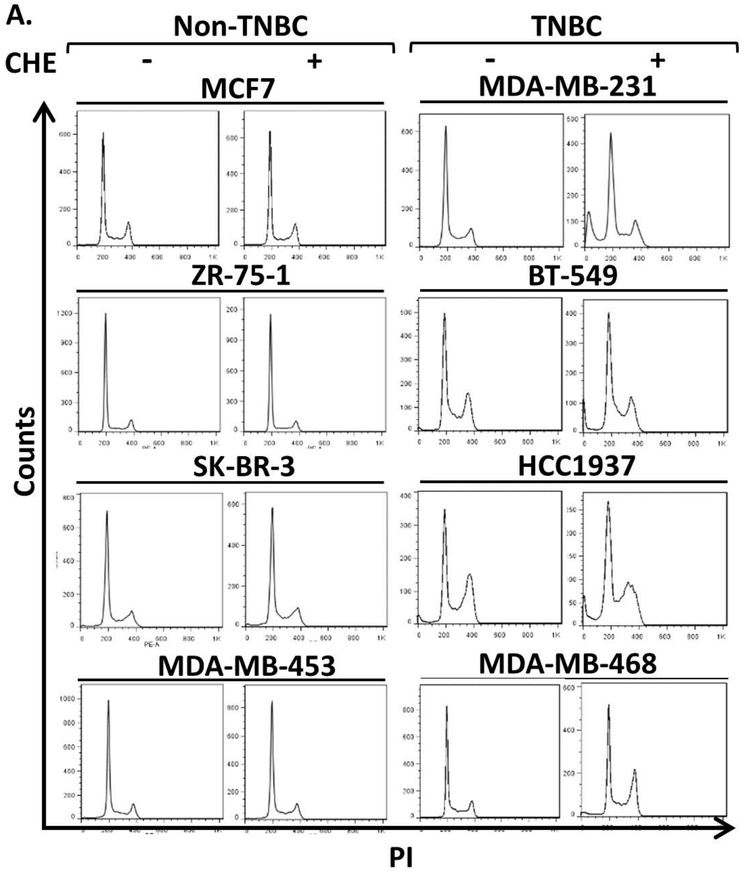

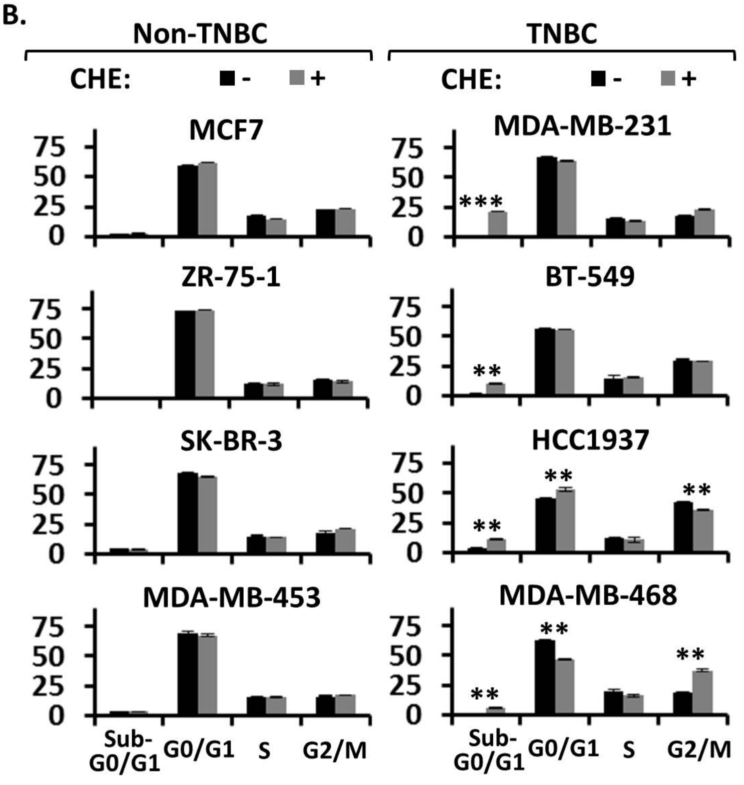

Supplementary Figure 2.

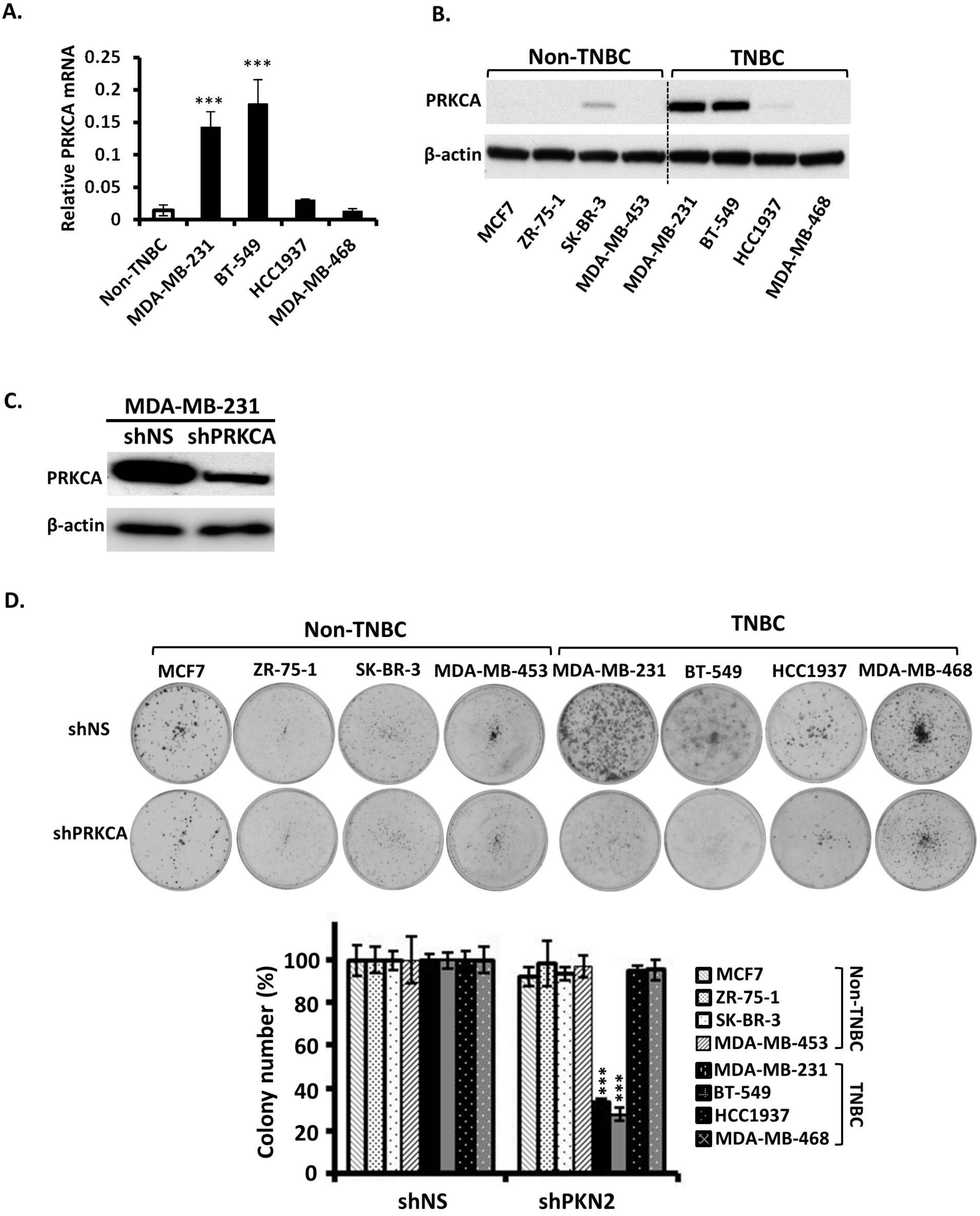

Supplement: Supplementary file 1 — Supplementary Information [file 41598_2017_2222_MOESM1_ESM.pdf]
